# Survival, growth, and potential of horizontal gene transfer of antibiotic resistance genes of bovine *Salmonella* on the cantaloupe surface

**DOI:** 10.1128/aem.01076-26

**Published:** 2026-10-01

**Authors:** Zaira L. Castro-Delgado, Norma Heredia, Jose Angel Merino-Mascorro, Eduardo Franco-Frías, Jorge E. Dávila-Aviña, Santos García

**Affiliations:** 1 Departamento de Microbiología e Inmunología, Universidad Autónoma de Nuevo León 27771, San Nicolás de los Garza, México; Michigan State University, East Lansing, Michigan, USA

**Keywords:** *Salmonella*, horizontal gene transfer, cantaloupe, food safety

## Abstract

*Salmonella* outbreaks are often linked to contaminated cantaloupes, and bacterial multiple antibiotic resistance is frequently observed. Research is needed to understand the growth and survival of *Salmonella* on cantaloupe surfaces, and the mechanisms of antibiotic resistance in that environment. This study investigated the growth, survival, and capacity for horizontal gene transfer of antibiotic resistance genes in *Salmonella* on the surface of cantaloupe. Bovine-derived *Salmonella* isolates were inoculated onto the surface of cantaloupe and incubated at 35°C under 65–75% humidity for 5 days. Each isolate was co-inoculated with donor *E. coli* CSH56 harboring helper plasmid RP4-8 and mobilizable plasmid pSC27, which carries kanamycin, neomycin, and ampicillin resistance genes. One hundred microliters of the *Salmonella* suspension (1 × 10^8^ CFU/mL) and 100 µL of the *E. coli* suspension (1 × 10^7^ CFU/mL) was applied to each cantaloupe (1:10 ratio). *Salmonella* survival and growth were monitored on Hektoen agar, transconjugants were enumerated on Hektoen agar with kanamycin, and donor *E. coli* were quantified on MacConkey agar. All *Salmonella* isolates survived and exhibited growth within the 5-day period, with increases ranging from 0.94 log_10_ CFU/mL to 3.43 log_10_ CFU/mL. Notably, all *Salmonella* isolates possess the capability to accept and integrate sequences derived from the pSC27 plasmid from *E. coli* into their chromosome, both *in vitro* and on cantaloupe. After conjugation, the count of *Salmonella* mutants carrying the plasmid was 2.79 log_10_ CFU/mL *in vitro*. The survival, growth, and horizontal gene transfer of *Salmonella* on the surface of cantaloupe pose significant risks to public health.

**IMPORTANCE:**

*Salmonella*, a leading cause of foodborne diseases, poses a global health threat, with multidrug resistance emerging as a severe problem. Cantaloupes have been implicated in salmonellosis outbreaks in multiple countries over the last three decades. We hypothesize that acquisition of antibiotic resistance genes from other bacteria may occur on the surface of the fruit. This study established that *Salmonella* can survive, proliferate, and acquire antibiotic resistance genes on cantaloupe surfaces. This observation underscores the potential risks on the growth and transfer of genes between pathogens such as *Salmonella* and *E. coli* in foods. This impact goes beyond disease production and contributes to the rapid escalation of antimicrobial resistance and transmission of virulence genes in foods and the environment, which is a significant threat to public health.

## INTRODUCTION

*Salmonella* is a leading cause of foodborne gastroenteritis in humans worldwide. Belonging to the *Enterobacteriaceae* family, it comprises over 2,600 serovars, including the serotypes Enteritidis and Typhimurium, which are the two most important serotypes of *Salmonella* transmitted from animals to humans in most parts of the world (1). Annually, approximately 21.7 million cases of *Salmonella* infection are reported worldwide, resulting in 216,000 fatalities. Notably, medium- and low-resource developing countries are disproportionately affected by this issue (2).

*Salmonella* infections are typically associated with the consumption of contaminated food items, such as eggs and chicken. However, agricultural products such as fruits and vegetables also play a role in the transmission of this infection (2). In 2023, the CDC reported 407 cases of disease in 44 states in the United States and Canada, resulting in six deaths. Cantaloupes have been implicated in numerous salmonellosis outbreaks in multiple countries over the last three decades (3). *Salmonella* contamination of cantaloupes during the production process can be influenced by various intrinsic and extrinsic factors (4).

Intrinsic factors include events such as biofilm formation and organic material in the cantaloupe surface that could promote a favorable environment for bacterial growth and survival (5). Extrinsic factors encompass sources of contamination such as soil, irrigation water, the use of organic fertilizers (which may originate from animal feces), contamination by feces from wild animals, livestock, or human waste, the temperature of the water used to wash the melons after harvest, and improper handling practices (5, 6).

The use of untreated bovine manure poses a significant risk of introducing pathogenic bacteria into agricultural soil. Research shows that *Salmonella* can persist in soil for 90 to over 200 days, contaminating cultivated products (7). While the FDA regulates the use of organic fertilizers containing manure in horticultural crops, feces can still be dispersed through irrigation water contaminated by agricultural runoff (8).

Fecal indicator bacteria have been demonstrated to survive and proliferate on the cantaloupe’s surface stored at 10°C with 95% relative humidity for a period of 20 days (9). Furthermore, *Salmonella* has been shown to proliferate on the peel up to 2.5 log_10_ CFU/cm^2^ after 24 h of incubation at room temperature (3, 10).

Cantaloupe fruit contamination can occur through direct contact of the pathogen with the surface of the fruit, leading to internalization of the pathogen to the internal pulp. The rough, flake-like texture of the outer structure of this fruit facilitates bacterial adhesion and biofilm formation on its surface, rendering decontamination of the rind difficult (11). This contributes to the persistence of microorganisms on the fruit (3, 10).

The emergence of multidrug resistance in foodborne pathogens such as *Salmonella* is a severe problem in food safety and human health. This organism is on the list of high-priority pathogens for antimicrobial resistance, according to the WHO. Horizontal gene transfer (HGT) facilitates the acquisition of antibiotic resistance genes (ARGs) among bacteria, enabling them to acquire novel genes with novel functionalities (12). This process plays a crucial role in the evolution of bacterial genomes, leading to population diversification, emergence of novel phenotypes, and impacts on antibiotic resistance, virulence, and compatibility with environmental stress (13, 14).

The mechanisms of HGT are mediated by mobile genetic elements, including plasmids, transposons, and bacteriophages. Integrons, although not mobile genetic elements themselves, are platforms that capture and express antimicrobial resistance gene cassettes. Their horizontal dissemination depends on their association with mobile genetic elements such as plasmids and transposons, facilitating the capture and dissemination of antimicrobial resistance genes. Extracellular vesicles or exosomes have also been reported to participate in HGT (12).

The HGT process can occur through three principal mechanisms: (i) transduction, mediated by phages; (ii) transformation, primarily involving the uptake of free DNA from the environment; and (iii) conjugation, characterized by the unilateral transmission of DNA from one cell to another via “sexual pilus,” with plasmids being the primary contributor. Recently, the mechanism of transfer by vesicles has also been investigated (12). Among these mechanisms, conjugation is considered the primary promoter of gene exchange, as well as the capture of free DNA by transformation (14).

During the pre-harvest, harvest, and postharvest stages of cantaloupe, it is exposed to various conditions and activities that facilitate the contamination, survival, growth, and interaction of different bacteria on the fruit. HGT is a process associated with the acquisition of novel capabilities within bacterial cells, and limited information is available regarding the conditions that facilitate its occurrence (12, 14). We hypothesize that HGT may occur on the surface of the fruit, as evidenced by the prolonged survival and growth of bacteria on the cantaloupe surface. Given the limited information available on the mechanisms of pathogen colonization and persistence on cantaloupe surfaces, and the insufficient data on the transfer of genomic material in these instances, this study aimed to investigate the capacity of *Salmonella* to survive and proliferate on cantaloupe surfaces and determine the feasibility of horizontal gene transfer (HGT) of antibiotic resistance genes from *Escherichia coli* to *Salmonella* under these conditions.

## MATERIALS AND METHODS

### Bacteria, plasmids, and growth conditions

The *Salmonella enterica* ser Typhimurium ATCC 14028 (kindly donated by Becton Dickinson Co., Mexico) was used as a positive control. The isolates *Salmonella* 5.2 (BC-NL-5.2), *Salmonella* 20.2 (BC-AG-20.2), and *Salmonella* 28.2 (BC-NL-28.2) were isolated from bovine feces in Mexico by our lab personnel; all these isolates were kanamycin-sensitive. *E. coli* CSH56 (donor strain) harboring plasmid RP4-8 gentamicin resistant (Gen^r^) was obtained from Addgene, USA (Michael Hynes Addgene plasmid #79813). Subsequently, it was transformed by electroporation with the plasmid pSC27 (Judy Wall, Addgene plasmid #117478), which confers kanamycin resistance (Kan^r^) and was used for the conjugation experiments. The isolates were kept in independent stock cultures at −80°C in ICC broth with 20% glycerol. Fresh cultures were obtained by transferring an aliquot in 5 mL of ICC incubated at 37°C for 24 h.

### Preparation of the cantaloupe surface

Cantaloupes were obtained from supermarkets within the metropolitan region of Monterrey, Mexico. These were selected with good appearance and absence of any discernible or apparent damage, at a state of ripeness suitable for consumption. The fruits were gently washed with liquid soap (Likofen, Chizen & Zen S.A. de C.V, México) at 5 mL/L in water for 5 min over the entire surface (ensuring that the entire surface of the fruits was thoroughly cleaned without causing any physical harm). The fruits were then placed in sterile Nasco Whirl-Pak bags and immersed in a 200 ppm chlorine solution (Chloralex, Grupo Alen. México) for 5 min with gentle shaking. Chlorine was removed by three rinses with sterile distilled water, and the melons were then immersed in sterile Nasco Whirl-Pak bags containing sterile distilled water for 1 min with gentle shaking. Cantaloupes were dried for 60 min in a laminar flow chamber at room temperature (25°C). Finally, the fruits were exposed to UV-C light (MEL, México) 200–280 nm for 10 min. Seven 5 × 5 cm areas were delineated within the melon, each corresponding to a specific time point (days) for monitoring (0, 1, 2, 3, 4, 5). An area with similar dimensions but without inoculation served as a negative control. This was undertaken to ascertain the absence of *Salmonella* contamination that was not directly related to the experiment.

### Cantaloupe inoculation and storage conditions

Bovine-derived *Salmonella* strains were individually inoculated onto the surface of cantaloupe at a concentration of 1 × 10^8^ CFU/mL in 10 μL volumes for each monitored time period. These volumes were inoculated using the dot-drop technique in several delineated areas measuring 5 × 5 cm. The inoculated fruits were left at room temperature for 1 h to allow the inoculum to dry and were then incubated at 35°C with 65–75% RH for 5 days in an SL SHEL Lab incubator (Sheldon Manufacturing, USA).

### Growth and survival of *Salmonella* on the surface of cantaloupe

On each day (0–5 days), the survival and growth of *Salmonella* on the surface area of the cantaloupe were quantified. For this purpose, a swab previously dipped in 1 mL of 0.1% alkaline peptone water was rubbed against the inoculated area for 2 min. Subsequently, the swab was swabbed back into the alkaline peptone water for another 2 min to release the bacterial cells. The samples were plated in duplicate on Hektoen agar, incubated at 37°C for 24 h, and colonies with black centers characteristic of this microorganism were enumerated. In addition, the presence of *Salmonella* was confirmed by PCR using the oligonucleotides encoding the STM3098 genes (15) with the primers 5′-TTTGG CGGCG CAGGC GATTC-3′ forward and 5′-GCCTC CGCCT CATCA ATCCG-3′reverse with an amplification size of 423 bp, using the following amplification conditions: one cycle of 95°C for 2 min, followed by 35 cycles at 95°C for 30 s, 60°C for 30 s, and 72°C for 30 s, and one cycle at 72°C for 5 min. The PCR product was visualized by electrophoresis on 1.5% agarose gel stained with GelRed Nucleic Acid Gel Stain (1×) and visualized under UV illumination.

### Construction of the donor strain in conjugation experiments (*E. coli* CSH56 with RP4-8 and pSC27 plasmids)

The *E. coli* CSH56 strain was obtained from Addgene. The kanamycin-resistant (Kan^r^) conjugative plasmid pSC27 (8,574 bp) was introduced into this strain via electroporation. For the transformation experiment by electroporation, the *E. coli* cells were adjusted to an OD of 0.5 at 600 nm and washed with cold Milli-Q water. From this suspension, 40 µL was taken, and 3 µL of the previously extracted pSC27 plasmid (400 ng) was added; both were introduced into an electroporator (Eppendorf model Eporator, Germany) at a voltage of 1,700 kV with a time constant of 5 ms, according to the indications of the general protocol.

The transformed cells were recovered with 1 mL of SOC broth and placed in a shaker (Thermo Scientific MaxQ mini 4450) at 170 rpm for 1 h at 37°C, and then inoculated on Luria-Bertani agar (Neogen, USA) supplemented with 10 µg/mL gentamicin (BIO Basic product, USA) and 50 µg/mL kanamycin (Sigma-Aldrich, USA) (LB^Gen+Kan^), and incubated at 37°C for 24 h. To confirm transformation, plasmids from *E. coli* were isolated through alkaline lysis and visualized on a 1% agarose gel stained with GelRed Nucleic Acid Gel Stain (1×) under UV illumination.

### Inoculum for conjugative experiments

Fresh cultures of the donor *E. coli* CSH56(pSC27+RP4-8) strain were obtained in 10 mL of Luria-Bertani broth (LB, Neogen, USA) supplemented with 10 µg/mL gentamicin and 50 µg/mL kanamycin (LB^Gen+Kan^) and incubated at 37°C overnight. In the case of *Salmonella*, it was grown in 10 mL of LB broth without antibiotics at the same conditions. Cultures were then centrifuged at 3,500 rpm for 10 min, washed three times with 5 mL of LB Lennox without antibiotics, and finally resuspended in 1 mL of LB Lennox without antibiotics. Bacterial concentration was adjusted to an OD_600_ of 0.5.

### *In vitro* horizontal transfer of pSC27 plasmid from *E. coli* CSH56 to *Salmonella*

The *in vitro* horizontal transfer capacity between *E. coli* and *Salmonella* isolates was studied. Fresh cultures of *E. coli* CSH56(pSC27+RP4-8) and *Salmonella* isolates were prepared. The cultures were centrifuged and washed three times with LB Lennox broth. A mixture of bacteria containing 1 × 10^7^ CFU/mL of donor *E. coli* and 1 × 10^8^ CFU/mL of recipient *Salmonella* (1:10 proportion) was prepared and incubated for 1 h and 24 h at 37°C. Formed transconjugants were quantified by serial diluting the culture and spreading it on Hektoen agar supplemented with 50 µg/mL kanamycin (Hektoen^Kan^) by duplicate.

Transconjugants were confirmed through PCR using two distinct oligonucleotides. One corresponded to the *r* sites originating from the replication site of the pSC27 plasmid to determine the integration of this plasmid into bacteria. The primers for this oligonucleotide were designed to amplify a 287-bp fragment 5′-CAATACGCAAACCACCGCCTCTCTC-3′ forward and 5′-CGGCCTTCGGTCATTACGTA-3′ reverse. The second oligonucleotide was specific for the kanamycin resistance gene in the pSC27 plasmid. The Kan gene, which contains a 300 bp resistance coding region in the plasmid, was amplified using the primers 5′-GCAGGATCTCCTGTCATCTC-3′ forward and 5′-TCCACCATGATATTCGGCAA-3′ reverse.

The following PCR amplification conditions were used for both plasmid-specific target genes: an initial denaturation at 95°C for 2 min, followed by 35 cycles of 95°C for 30 s, 56°C for 30 s, and 72°C for 30 s, with a final extension at 72°C for 5 min. PCR products were visualized by electrophoresis on a 1.5% agarose gel stained with GelRed Nucleic Acid Gel Stain (1×) and visualized under UV illumination.

### Horizontal transfer of pSC27 plasmid from *E. coli* CSH56 to *Salmonella* on cantaloupe surface

Isolate of *E. coli* as donor and *Salmonella* as receptor were inoculated on the cantaloupe surface as stated before. From the inoculated areas on the melon surface, swabs were collected as described above at 1 h and 24 h. Subsequently, aliquots of 200 µL were serially diluted and plated by extension on Hektoen agar supplemented with 50 µg/mL kanamycin (Hektoen^Kan^) in duplicate and incubated at 37°C for 24 h. Transconjugated colonies exhibiting a black center, indicative of *Salmonella*, were quantified. Transconjugants were confirmed by PCR analysis of the presence of the *Kan* gene and the origin of replication site of the pSC27 plasmid.

### Genome sequencing (WGS)

In accordance with the higher number of bacterial transconjugant isolates obtained on the surface of cantaloupe, isolates BC-NL 5.2 and *S*. Typhimurium ATCC 14028, along with their respective transconjugants, were selected for whole-genome sequencing utilizing Oxford Nanopore technology.

DNA extraction was performed in accordance with the protocol of QIAamp using the QIAamp DNA Mini Kit (Qiagen, Germany; Cat. No. 51304). The manufacturer’s instructions were followed with minor modifications, including the addition of RNase A for RNA-free samples. Three column centrifugations were performed at the conclusion of the procedure using RNA-free water. DNA was eluted using nuclease-free water. DNA quality was evaluated using the NanoDrop 2000 spectrophotometer (Thermo Fisher Scientific, USA) and quantified using the Qubit fluorometer (Thermo Fisher, USA). The Nanopore sequencing library was generated using the Rapid Barcoding SQK-RBK004 Kit following the manufacturer’s instructions. The library was sequenced using the FLO-MIN106D flow cell (R9.4.1) integrated with the MinION device.

### Sequence analysis

Whole-genome sequencing was performed using the Oxford Nanopore Technologies (ONT) MinION platform operated with MinKNOW (version 25.05). Real-time base calling analysis was carried out using the base-calling algorithm Dorado (Oxford Nanopore Technologies, version 1.1.0). The analysis was performed using EPI2ME software (Oxford Nanopore Technologies, ONT, 2024 version 25.05), driven by the wf-bacterial-genomes workflow (version 1.4.2). Adapter trimming and quality assessment were analyzed by NanoPlot (version 1.46.1) (16).

High-quality long reads were *de novo* assembled using Flye (version 2.9.5) with parameters optimized for ONT long-read sequencing. The assembled genomes were subsequently polished using the Medaka workflow implemented in EPI2ME. Genome annotation was performed using Prokka (version 1.15.6) (17). Chromosomal contigs were reordered relative to the reference genome using Mauve genome alignment software (version 2.4.0), and the annotated assemblies were further inspected and curated using Geneious Prime (version 2025.2.1) (https://www.geneious.com).

Whole-genome relatedness was assessed by comparing the genomic sequences of *Salmonella* isolates and transconjugants with reference genomes representing different *Salmonella* species and serovars, using orthologous average nucleotide identity (OrthoANI) implemented in the OAT software (18). Pairwise OrthoANI values were calculated to evaluate genomic similarity and confirm species-level taxonomic assignment using the widely accepted threshold of ≥95–96% average nucleotide identity (ANI).

Serotype assignment and genomic characterization of strain BC-NL5.2 were performed by *in silico* analysis of the assembled genome in FASTA format using the sistr_cmd tool (*Salmonella In Silico* Typing Resource, v1.1.3) integrated into the Use Galaxy Europe public server (https://usegalaxy.eu). Serovar prediction was obtained from the antigenic formula (O, H1, and H2 antigens) and core genome sequence typing (cgMLST, 330 loci) (19). To position our isolated strains within the context of relevant *Salmonella enterica* reference genomes retrieved from GenBank, a phylogenetic tree based on genomic distances was constructed. The methodology and resulting phylogenetic tree are provided in Fig. S1.

The horizontal transfer of the pSC27 plasmid from the donor *E. coli* strain to *Salmonella* isolates recovered from cantaloupe surfaces was initially evaluated by comparing the complete genomic assemblies of recipient isolate before and after conjugation on cantaloupe. Evidence of plasmid acquisition was further assessed through comparative genomic analyses in Geneious Prime, including BLAST searches against the assembled genomes using the complete pSC27 sequence as the reference.

Candidate chromosomal integration events were identified by detecting plasmid-derived genes and contiguous plasmid-specific genomic regions within the recipient chromosome. The insertion boundaries and genomic organization of the integrated fragment were manually inspected and visualized using SnapGene (Version 8.1.1. SnapGene software from Dotmatics; available at https://www.snapgene.com/).

### Statistical analysis

**Fig 1:**
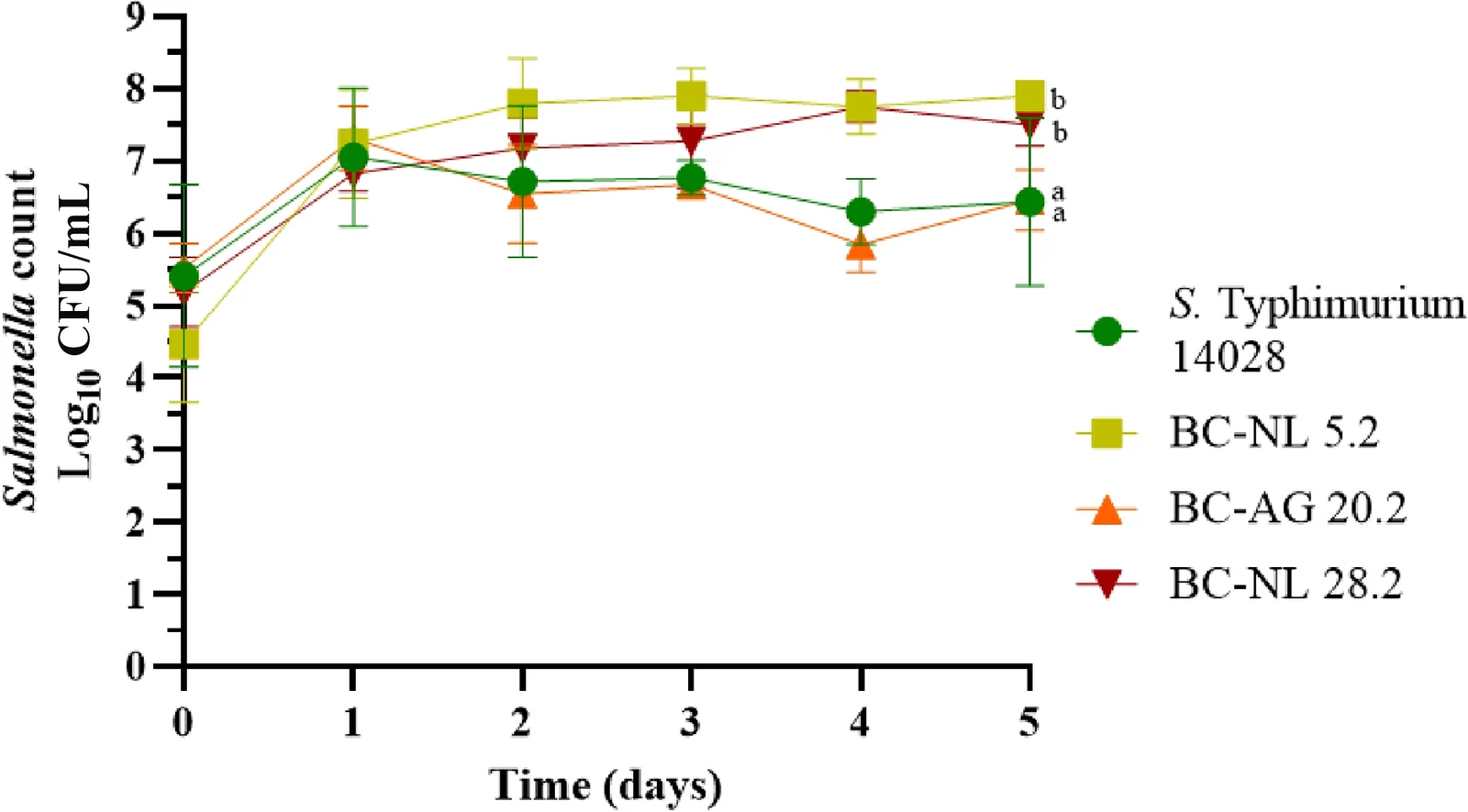
Survival and growth of *Salmonella* isolates on the surface of cantaloupe at 35°C (RH, 65–75%). Control: *S*. Typhimurium ATCC 14028. Different letters represent differences between the means of the strains. The bars represent the mean ± standard deviation (*n* = 6).

**Fig 2:**
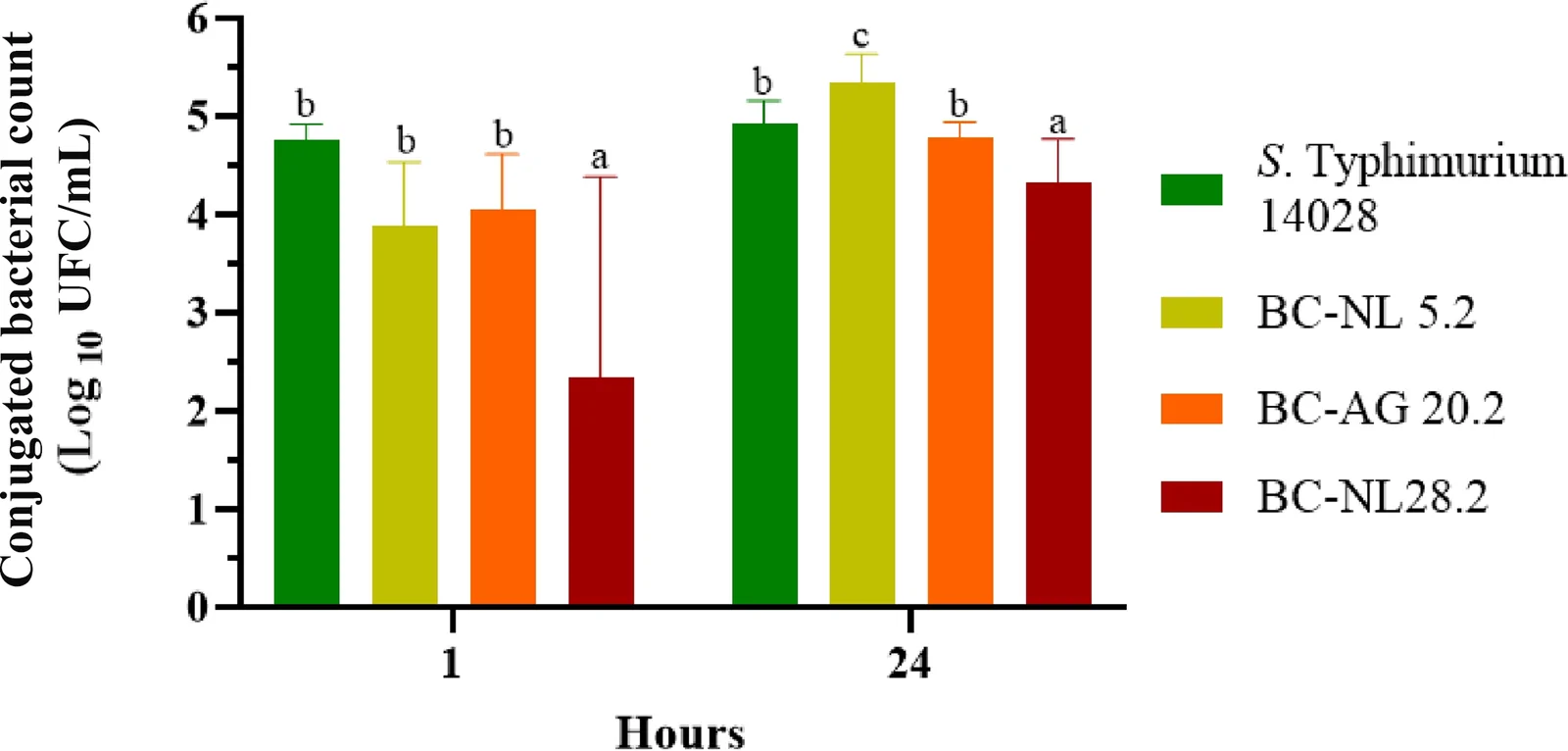
*In vitro* conjugation of *Salmonella* isolates using *E. coli* CSH56(pSC27+RP4-8) as the donor strain. Control receptor: *S*. Typhimurium ATCC 14028. Different letters represent differences between the means of the strains. The bars represent the mean ± standard deviation (*n* = 6).

**Fig 3:**
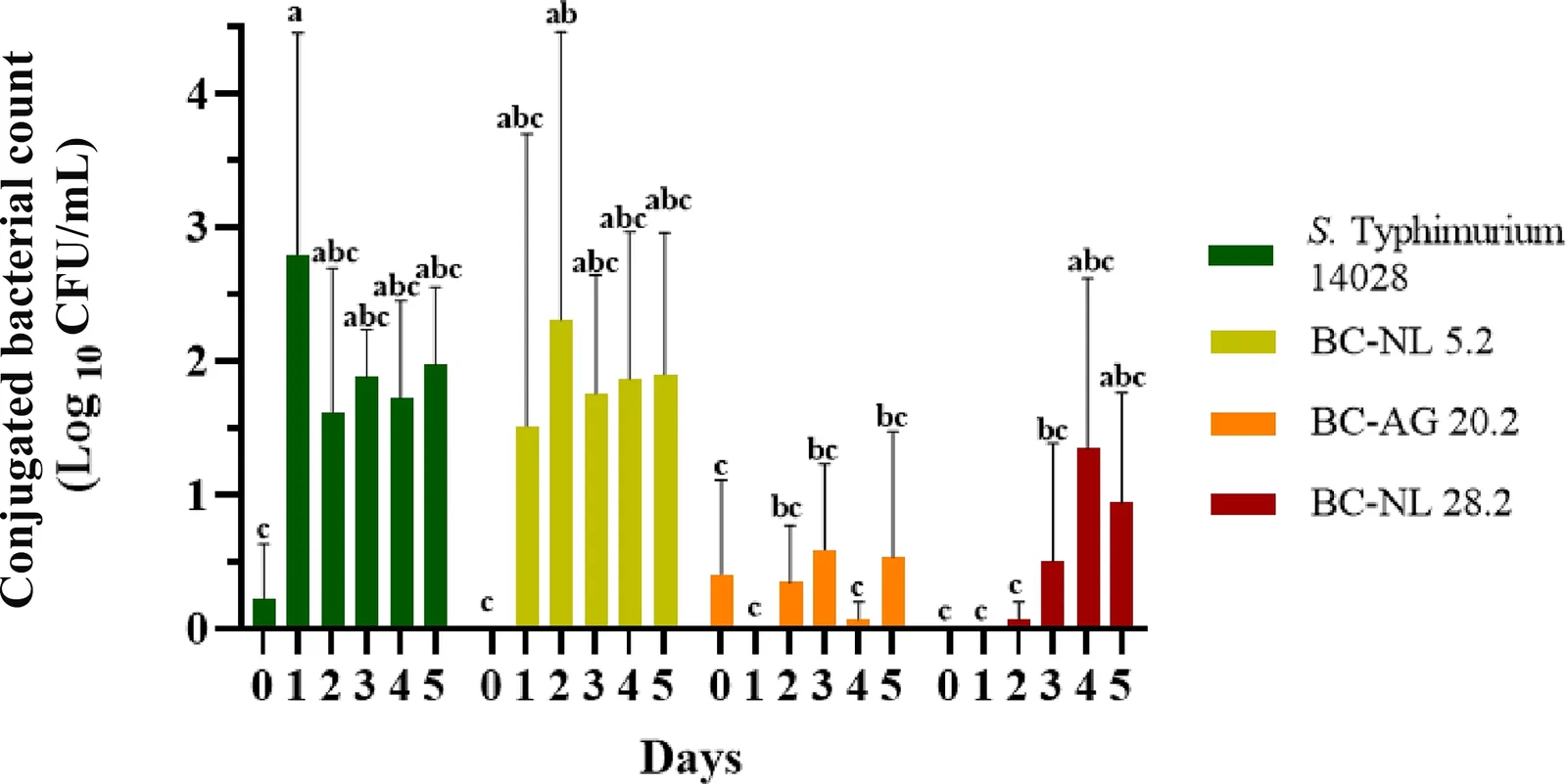
*In situ* horizontal transfer of pSC27 plasmid from *E. coli* CSH56 to isolates of *Salmonella* on the surface of cantaloupe stored at 35°C and 65–75% relative humidity for 5 days. The bars represent the mean ± standard deviation. Different letters above the bars indicate statistically significant differences (*P* < 0.05) among treatment combinations.

The experiment was conducted with three biological replicates, each analyzed in technical duplicate (*n* = 6). Data were analyzed using analysis of variance (ANOVA). Dunnett’s post hoc test was applied to compare experimental treatments against the control group for Fig. 1 and 2. In contrast, Duncan’s post hoc test was used for pairwise comparisons among all treatment groups for Fig. 3. Statistical significance was established at *P* < 0.05. All statistical analyses were conducted using IBM SPSS Statistics for Windows, Version 26.0 (IBM Corp., Armonk, NY, USA), and figures were generated using GraphPad Prism version 9.5.0 (GraphPad Software, San Diego, CA, USA).

## RESULTS

### Growth and survival of *Salmonella* on the cantaloupe surface

The growth and survival of *Salmonella* strains on the cantaloupe surfaces were analyzed using ANOVA, considering the bacterial isolate, time, and their interaction (time-bacteria *P* < 0.05). This analysis revealed that population changes over the observation period were strain-dependent.

The initial concentration of *Salmonella* isolates on the cantaloupe surfaces was 5.4 log_10_ CFU/mL for *S*. Typhimurium, 5.18 log_10_ CFU/mL for isolate BC-NL 28.2, 4.46 log_10_ CFU/mL for BC-NL 5.2, and 5.51 log_10_ CFU/mL for BC-AG 20.2. Growth was observed in all *Salmonella* isolates throughout the time period (Fig. 1), with the greatest increase at 24 h post-inoculation. At this time, BC-NL 5.2 exhibited the most pronounced growth, resulting in a 2.76 log_10_ CFU/mL increase. BC-AG 20.2 exhibited a 1.79 log_10_ CFU/mL increase, while *S*. Typhimurium and BC-NL 28.2 also exhibited growth, albeit with a smaller increase of 1.64 log_10_ CFU/mL for both isolates.

Over the subsequent days of monitoring, the growth and survival of all *Salmonella* isolates persisted (Fig. 1). Isolate BC-NL 5.2 showed a higher population increase compared to the other isolates. BC-NL 28.2 reached its peak population on day 4. BC-AG 20.2 exhibited a substantial increase within the initial 24 h, followed by a gradual decline. Nevertheless, its concentration consistently exceeded the initial level. Throughout the 5-day study period, all *Salmonella* isolates maintained levels exceeding the initial inoculum. At the end of the 5-day incubation period on the cantaloupe surface, the population increases of the isolates were as follows: *S*. Typhimurium, 1.02 log_10_ CFU/mL; BC-NL 5.2, 3.43 log_10_ CFU/mL; BC-AG 20.2, 0.94 log_10_ CFU/mL; and BC-NL 28.2, 2.31 log_10_ CFU/mL. This indicates that all *Salmonella* isolates were capable of surviving and growing on the melon surface, where the BC-NL 5.2 isolate showed the greatest increase in growth and survival.

Comparisons with the control group (*S*. Typhimurium 14028) revealed that strain BC-AG 20.2 exhibited average population levels comparable to those of the control. In contrast, isolates BC-NL 5.2 and BC-NL 28.2 differed in average means from the *S*. Typhimurium 14028.

### *In vitro* conjugation of pSC27 plasmid from *E. coli* CSH56 to *Salmonella*

*In vitro* conjugation assays revealed that plasmid transfer exhibited significant differences based on both the recipient isolate (*P* < 0.05) and incubation time (*P* < 0.05), with no significant interaction between strain and time (*P* > 0.05) (Fig. 2).

All *Salmonella* isolates were conjugated by the insertion of the pSC27 plasmid from *E. coli* after 1 and 24 h (Fig. 2). At 1 h of incubation time, BC-NL 5.2 and BC-AG 20.2 exhibited plasmid transfer rates comparable to *S*. Typhimurium. In contrast, BC-NL 28.2 showed a lower number of conjugated bacteria. After 24 h of incubation, BC-NL 5.2 had the highest number of conjugated bacteria. BC-AG 20.2 maintained transfer levels similar to *S*. Typhimurium, whereas BC-NL 28.2 exhibited a lower transfer.

In terms of conjugation frequency, the BC-NL 5.2 isolate exhibited superior performance across all assays (Table 1), demonstrating the highest capacity for DNA acquisition through HGT.

**TABLE 1:** *In vitro* conjugation frequency (CFU) of *Salmonella* in the presence of *E. coli* CSH56(pSC27+RP4-8) by HGT

*Salmonella* strain	Exposure time to 37°C (h)	Conjugation frequency in the presence of *E. coli^a^*
*S*. Typhimurium	1	1.93 × 10^−4^ ± 7.19 × 10^−4^
	24	2.92 × 10^−4^ ± 1.42 × 10^−4^
BC-NL 5.2	1	5.87 × 10^−5^ ± 8.13 × 10^−5^
	24	8.96 × 10^−4^ ± 5.92 × 10^−4^
BC-AG 20.2	1	1.10 × 10^−4^ ± 1.24 × 10^−4^
	24	3.67 × 10^−4^ ± 1.17 × 10^−4^
BC- NL 28.2	1	1.02 × 10^−5^ ± 2.97 × 10^−6^
	24	4.63 × 10^−5^ ± 2.83 × 10^−5^

^*a*^ Values are expressed as mean ± standard deviation (SD) from three independent biological replicates performed in technical duplicate (*n* = 6).

### *In situ* horizontal transfer of pSC27 plasmid from *E. coli* CSH56 to isolates of *Salmonella* on the cantaloupe surface

All *Salmonella* isolates demonstrated the capacity to harbor the pSC27 plasmid-derived sequences via mobilization following co-cultivation with *E. coli* as a donor at 35°C with a relative humidity of 65–75% on the surface of the cantaloupe (Fig. 3). The ANOVA demonstrated significant differences based on the bacterial isolate conjugation, time, and interaction between time and bacterial isolate.

The isolate-time interaction indicates that the growth kinetics differed over time among the evaluated isolates. Comparisons reconfirmed that the *S*. Typhimurium exhibited the highest number of transconjugants at 24 h (day 1; 2.79 log_10_ CFU/mL), which was higher than the values observed on the remaining sampling days for this strain. After day 1, transconjugant counts decreased and remained relatively stable through day 5. For isolate BC-NL 5.2, the highest conjugation was detected on day 2 (2.30 log_10_ CFU/mL). In contrast, strain BC-AG 20.2 showed consistently low conjugation frequencies throughout the experiment (<0.80 log ^10^ CFU/mL).

The BC-NL 28.2 strain generally exhibited lower conjugation frequencies to the other isolates. Transconjugant counts on days 0, 1, and 2 were lower than those of the *S*. Typhimurium. Although conjugation increased and peaked on day 3 (1.28 log_10_ CFU/mL), this value remained lower than that of the control *S*. Typhimurium on the corresponding sampling day.

Similarly, during the subsequent 24 h of conjugation, higher values were observed in *Salmonella* BC-NL 5.2 and *S*. Typhimurium (Table 2). In contrast, isolates BC-AG 20.2 and BC-NL 28.2 exhibited reduced conjugation frequency compared to the *S*. Typhimurium.

**TABLE 2:** Conjugation frequency (CFU) *in situ* on the cantaloupe surface of *Salmonella* in the presence of *E. coli* CSH56(pSC27+RP4-8) by HGT

Days post-inoculation	Conjugation frequency^*a*^			
	*S*. Typhimurium	BC-NL 5.2	BC-AG 20.2	BC-NL 28.2
0	5.26 × 10^−9^ ± 8.80 × 10^−9^	0	5.16 × 10^−8^ ± 1.25 × 10^−7^	0
1	4.19 × 10^−5^ ± 6.41 × 10^−5^	1.17 × 10^−5^ ± 1.81 × 10^−5^	5.16 × 10^−9^ ± 1.25 × 10^−8^	0
2	7.84 × 10^−7^ ± 1.18 × 10^−6^	2.11 × 10^−5^ ± 3.14 × 10^−5^	2.58 × 10^−8^ ± 3.03 × 10^−8^	1.85 × 10^−9^ ± 1.01 × 10^−8^
3	2.92 × 10^−7^ ± 1.18 × 10^−7^	6.50 × 10^−7^ ± 9.11 × 10^−7^	6.71 × 10^−8^ ± 1.19 × 10^−7^	3.70 × 10^−8^ ± 1.28 × 10^−7^
4	4.05 × 10^−7^ ± 6.83 × 10^−7^	1.56 × 10^−6^ ± 2.53 × 10^−6^	5.16 × 10^−9^ ± 1.25 × 10^−8^	4.07 × 10^−7^ ± 1.22 × 10^−6^
**5**	4.50 × 10^−7^ ± 3.04 × 10^−7^	1.25 × 10^−6^ ± 2.37 × 10^−6^	1.29 × 10^−7^ ± 2.72 × 10^−7^	5.93 × 10^−8^ ± 1.28 × 10^−7^

^*a*^ Values are expressed as mean ± standard deviation (SD) from three independent biological replicates performed in technical duplicate (*n* = 6).

Overall, these results demonstrated that conjugation efficiency was influenced by both the bacterial isolate and the incubation time on the cantaloupe surface. While the *S*. Typhimurium exhibited the highest conjugation frequency during the first 24 h, the BC-NL 5.2 strain maintained a relatively high plasmid transfer efficiency over time. In contrast, the BC-AG 20.2 and BC-NL 28.2 strains showed reduced conjugation frequencies during most of the storage days.

### Identification of horizontal gene transfer of pSC27 plasmid-derived sequences in recipient isolates

**Fig 4:**
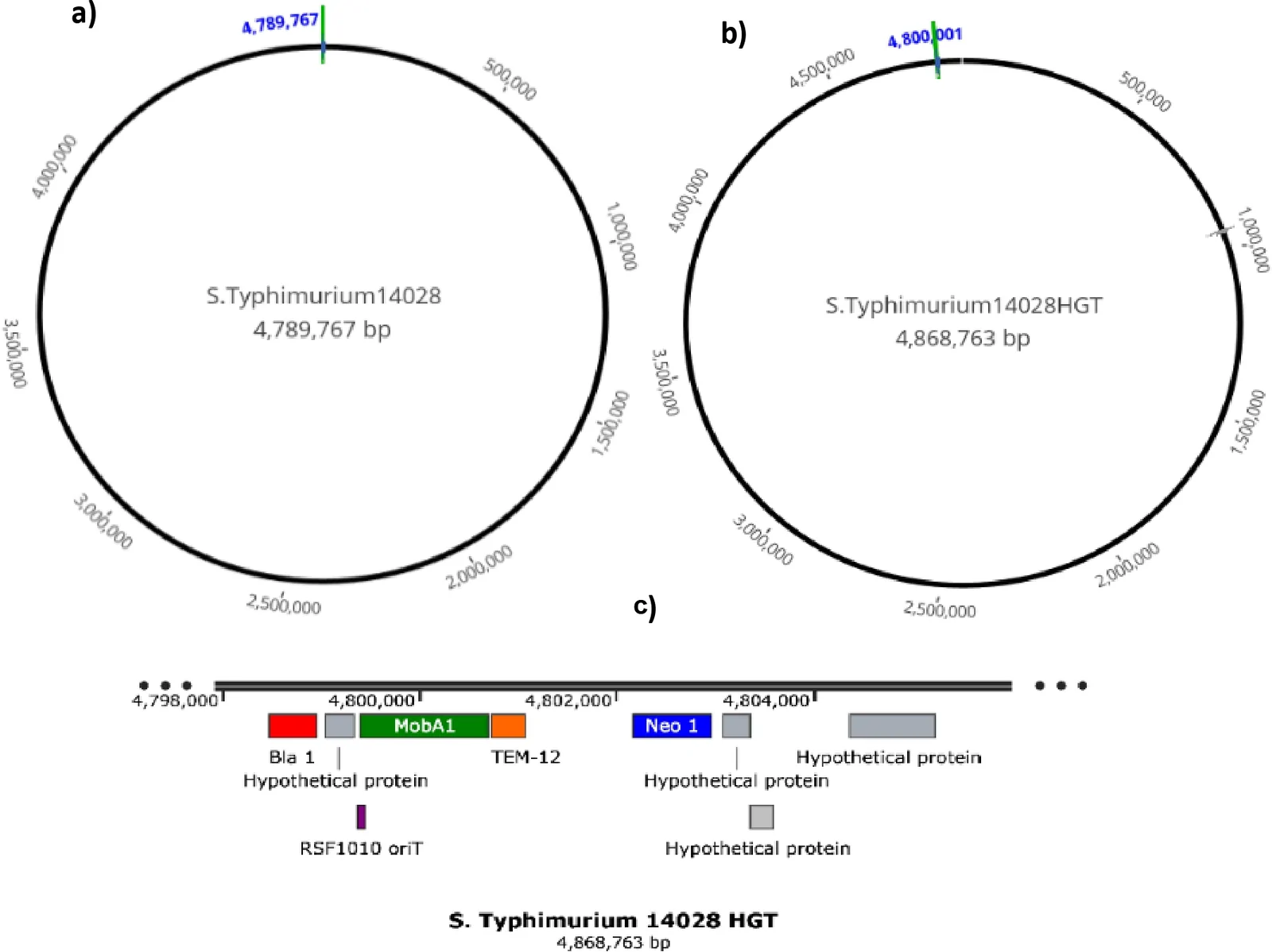
(**a**) Circularized genome of *S*. Typhimurium prior to conjugation, (**b**) Circularized transconjugant *S*. Typhimurium genome, and (**c**) Integrated fragment into the transconjugant *S*. Typhimurium chromosome from plasmid pSC27. Specific regions corresponding to fragments of hypothetical proteins, the *MobA1* mobility region, and resistance regions *Bla 1*, *TEM*-12, *Neo1,* and the replicative origin RSF1010 are identified.

**Fig 5:**
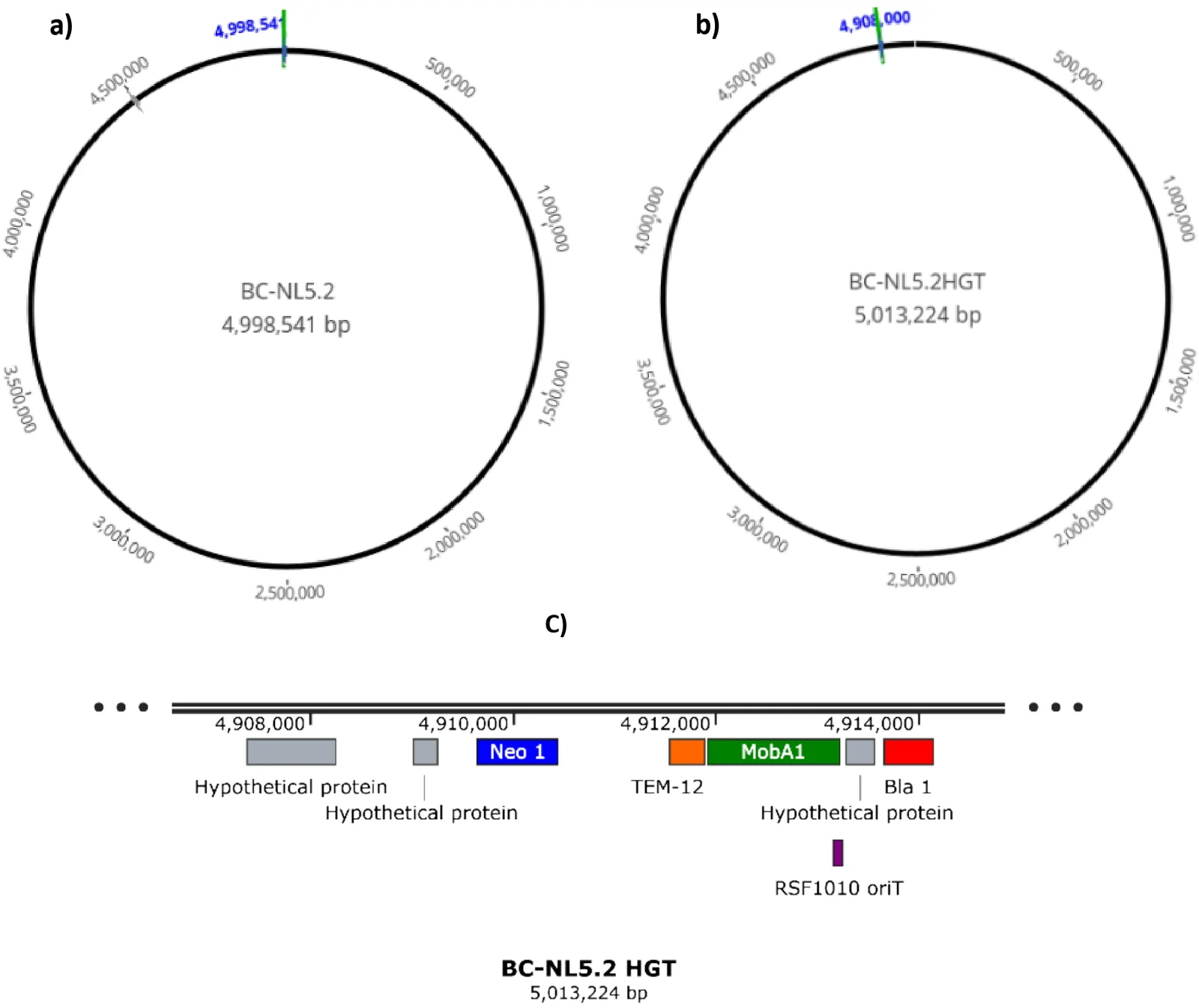
(**a**) Circularized BC-NL 5.2 genome without conjugation, (**b**) circularized transconjugant BC-NL 5.2 genome, and (**c**) fragment integrated into the transconjugant BC-NL 5.2 chromosome from plasmid pSC27. Specific regions corresponding to fragments of hypothetical proteins, the *MobA1* mobility region, and resistance regions *Bla 1*, *TEM*-12, *Neo1,* and the replicative origin RSF1010 are identified.

Whole-genome sequencing and comparative sequence alignment showed a distinct increase in genome size in the two transconjugant *Salmonella* isolates after exposure to the cantaloupe surface. *S*. Typhimurium 14028 HGT exhibited a genome size increase of 78,996 bp compared to the non-conjugating strain (increasing from 4,789,767 bp to 4,868,763 bp) (Fig. 4a and b). Similarly, an increase in the genome size of strain BC-NL 5.2 HGT was detected, with an increase of 14,683 bp (increasing from 4,998,541 bp to 5,013,224 bp) (Fig. 5a and b). Alignments confirmed that this structural expansion resulted from co-transfer and structural rearrangement of plasmid fragments. Both *Salmonella* transconjugant integrated sequences derived from the pSC27 plasmid, thus confirming the horizontal transfer event through conjugation.

Plasmid-derived sequences were detected in transconjugants, indicating the presence of genes from plasmid pSC27, an IncQ plasmid, on the cantaloupe surface. This was confirmed through whole-genome sequencing of *S*. Typhimurium and BC-NL 5.2. These genes were detected in both recipient *Salmonella* isolates after exposure to the donor strain *E. coli* CSH56(pSC27+RP4-8). More precisely, genes from the pSC27 plasmid were identified in both transconjugant isolates (Fig. 4c and 5c). The insertion of coding regions of the transferred plasmid, which are known as hypothetical proteins of the mobile elements of the plasmid in their functional domains, was determined by CD-Search of the MobA mobilization operon. Additionally, the corresponding antibiotic resistance of the *bla1*, *beta-lactamase TEM-12*, and *neo1* genes was assessed. Furthermore, the specific mobility and integration region of the OriT replication origin within the 88-bp RFS1010 region (specific for transmissibility) was identified (Fig. 4c and 5c).

Additionally, transconjugants were confirmed by PCR targeting a 287-bp fragment within *r* sites associated with the pSC27 plasmid replication/integration machinery. This confirmed the acquisition and accommodation of pSC27-derived sequences in *Salmonella* recipient strains.

### Genome-wide similarity analysis using the OrthoANI algorithm and taxonomic classification of *S*. Typhimurium 14028, BC-NL 5.2, and their transconjugants

*In silico* serotype prediction demonstrated high analytical reliability, achieving 100% core genome coverage with the identification of all 330/330 loci of the cgMLST330 scheme in the SISTR quality control module, significantly exceeding the minimum QC threshold established at 297 loci (19). This confirmed the taxonomic assignment of isolate BC-NL 5.2 to genus (*Salmonella*), species (S. *enterica*), subspecies (Subsp. *enterica*), and serovar (ser. Cerro).

The assessment of genetic relationships and divergence was conducted by analyzing average nucleotide identity (ANI), based on DNA similarity of aligned reference genome fragments. All sequences were analyzed using the NCBI database. Species-level similarity was defined as >80%, while subspecies-level similarity required >98% (20).

**Fig 6:**
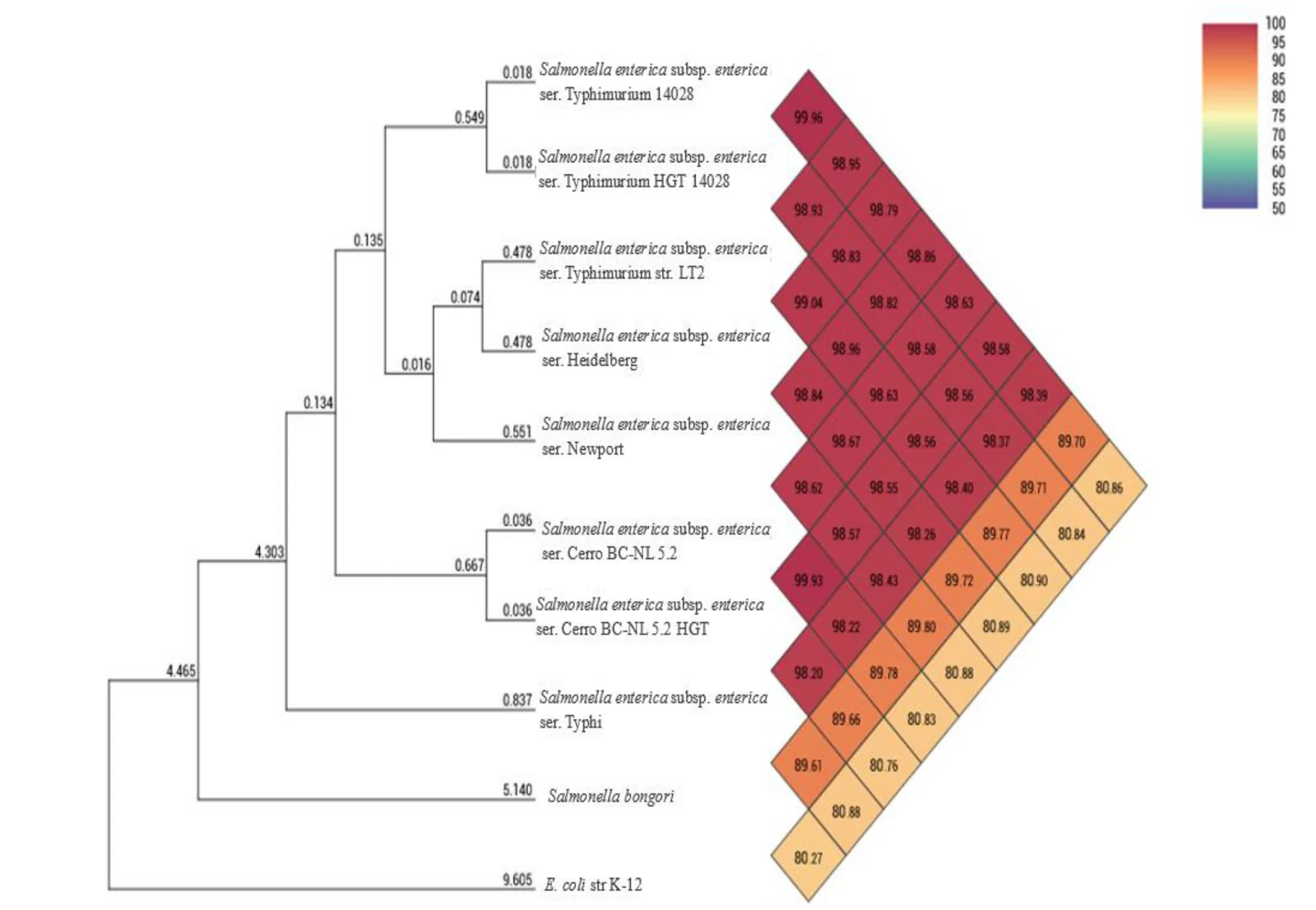
OrthoANI-based heatmap showing genetic relationships and divergence among *Salmonella* strains. The heat map illustrates pairwise orthologous average nucleotide identity (OrthoANI) values across *Salmonella*, categorizing them into various enteric species, subspecies, and serovar. Color intensity indicates sequence identity: red signifies the highest similarity (>98%), orange denotes moderate similarity (>89%), and yellow represents low similarity (<81%).

Genomic analyses were performed to determine genetic distance among species, enabling the construction of heat maps that corroborate lineage assignments using the OrthoANI program. In these heat maps, red indicates high similarity, orange indicates medium similarity, and yellow indicates low similarity. These analyses confirm the similarity of the isolates studied with the *Salmonella* species of concern in recent foodborne outbreaks (Fig. 6). Furthermore, to determine the genomic relationship of our isolates with relevant *Salmonella enterica* reference strains from GenBank, a phylogenetic tree was generated (Fig. S1). The isolated strains clustered closely with *Salmonella enterica* strains.

## DISCUSSION

The capacity of pathogens to adhere to the surfaces of fruits and vegetables presents a substantial concern for the agricultural industry and health authorities. Cantaloupe, which has been associated with outbreaks of foodborne illnesses, possesses a ridged rind network on its surfaces that features a mesh-like network of lenticels for gas exchange. This microenvironment creates a favorable condition for microbial adhesion and survival, rendering conventional sanitation methods ineffective for achieving comprehensive cleaning (21–23).

Horticultural crops such as melons are of tropical origin and typically grow in warm regions, with optimum growing temperatures ranging from 25°C to 35°C (77–95°F). As per the U.S. Food Code, foods such as cantaloupe must be stored at 5°C. The general guidance provided by the UCDAVIS Postharvest Technology Center for the postharvest handling and storage of cantaloupe recommends keeping them at a temperature range of 2.2°C to 5°C (36°F–41°F) for storage. However, higher temperatures such as 35°C are commonly encountered at various stages of the supply chain, including farm, picking, collection, storage, distribution, and retail, where microbial growth can occur (24, 25).

*Salmonella* from livestock farms is a significant source of infections, especially those with multidrug resistance (26). Sharing equipment or land between horticultural and cattle farms poses a risk of *Salmonella* dissemination. Common shared equipment includes tractors, trailers, wagons, plowing and harvesting machinery, and manure transport vehicles (27, 28).

The indiscriminate use of antibiotics in livestock, mainly as prophylactics and growth enhancers in non-EU regions, has led to global antibiotic resistance (29). While Europe banned antibiotic growth promoters in 2006, global veterinary consumption remains high. Prolonged exposure to low antibiotic concentrations increases selective pressure, facilitating resistance development through mechanisms like horizontal gene transfer (29).

*Salmonella* BC-NL 5.2 is the Cerro serotype, which is widely distributed in cattle. In the USA, epidemiological surveillance reports identify *S*. Cerro in over 16% of *Salmonella* isolates from dairy cattle (30). Likewise, in the context of antimicrobial resistance, although most *S*. Cerro isolates exhibit susceptibility profiles to conventionally used antibiotics, the presence of strains carrying mobile plasmids that confer resistance to tetracyclines, ampicillin, and sulfamethoxazole/trimethoprim has been documented (31). Similarly, this study identified its broad capacity to receive plasmids from other bacteria on the surface of the cantaloupe, making this strain a prominent genetic reservoir capable of horizontal gene transfer. Therefore, the characterization of strain BC-NL 5.2 like ser. Cerro underscores the importance of genomic surveillance in the food chain under the “One Health” approach.

Foodborne diseases caused by *Salmonella* are worsened due to the critical dissemination of antibiotic-resistance genes, changing the antibiotic susceptibility patterns of the bacterium and limiting the effectiveness of commonly used antibiotics (32). Livestock feces serve as a source of contamination not only for beef and beef-derived products but also for produce (33, 34). Cattle from the diverse regions of Mexico are reservoirs of multidrug-resistant bacteria (35).

The current crisis of antibiotic resistance underscores the paramount significance of comprehending the role of pathogens in their accelerated evolution through mechanisms such as HGT. The ability to acquire plasmids depends on the mechanism that the plasmid itself develops, where conjugative plasmids play a key role, as they contain all the genes necessary for their maintenance during gene transfer (36).

The influence of smooth or rough surfaces on bacterial growth and survival was studied using models adapted to these surfaces. Ordaz et al. (9) investigated the survival and growth of *E. coli* and *Enterococcus* on a smooth surface (associated with tomatoes) and a rough surface (associated with cantaloupes) over a 25-day period at two different temperatures and relative humidity (10°C/95% RH and 25°C/65% RH). The bacteria survived and grew during both periods, with the rough surface showing higher growth and survival rates.

Temperature significantly influences *Salmonella* persistence on fresh produce. Bacterial growth increases markedly as temperature approaches the physiological optimum (37°C), especially under high relative humidity (37). Bacterial cells grown at 37°C exhibit enhanced persistence after contamination of fresh produce, indicating improved bacterial fitness and survival (38). Cantaloupe supports *Salmonella* persistence and proliferation more effectively than other fruits and vegetables due to its nutrient-rich tissues and rough, reticulated rind, which retains moisture and provides protected microenvironments for bacterial survival (24, 39). Therefore, the present study uses 35°C, which closely approximates the optimal growth conditions for *Salmonella*. These results are especially relevant under current climate change scenarios, where rising temperatures may increase bacterial proliferation when refrigeration is insufficient. The FDA and CDC emphasize maintaining perishable foods at ≤4°C and refrigerating them promptly, as bacterial pathogens such as *Salmonella* multiply rapidly outside the recommended range.

The study evaluated conditions simulating temperature abuse during post-harvest handling, storage, or when cantaloupe is stored at home. Under standard commercial refrigeration (4°C, 95% RH), *Salmonella* spp. survive but cannot proliferate on cantaloupe surface (40). Compromised cold chain integrity allows higher ambient temperatures to overcome thermal inhibition, enable multiplication on cantaloupe surface, and maintain active metabolic processes facilitating plasmid horizontal transfer.

Of the four different *Salmonella* isolates analyzed in this study, all were able to survive and grow on the surface of melon at 35°C with 65–75% RH. Isolate BC-NL 5.2 showed the highest growth in the first 24 h (2.76 log_10_ CFU/mL), followed by isolate BC-AG 20.2 (1.79 log_10_ CFU/mL), *S*. Typhimurium (1.64 log_10_ CFU/mL), and isolate BC-NL 28.2 (1.64 log_10_ CFU/mL). The total increase in CFU/mL between day 0 and day 5 was 1.02 log_10_ for *S*. Typhimurium, 3.43 log_10_ CFU/mL for BC-NL 5.2, 0.94 log_10_ CFU/mL for BC-AG 20.2, and 2.31 log_10_ CFU/mL for BC-NL 28.2. At the conclusion of the 5-day analysis, it was determined that the BC-NL 5.2 isolate showed greater growth at the end of the 5-day analysis with a total count of 7.89 log_10_ CFU/mL. This was followed by isolate BC-NL 28.2 with 7.49 log_10_ CFU/mL, *S*. Typhimurium with 6.43 log_10_ CFU/mL, and isolate BC-AG 20.2 with 4.45 log_10_ CFU/mL.

*Salmonella* grew and survived on cantaloupe during the 5-day storage period, with strain-dependent growth. Isolate BC-NL 5.2 showed the greatest increase, while BC-AG 20.2 showed only a moderate increase, indicating strain variability in adaptation to the fruit surface. Harrand et al. (38) found that *Salmonella* persistence on fresh produce is influenced by bacterial physiology and environmental conditions. Bacterial growth is consistent with previous reports showing that temperatures near the optimum for *Salmonella* (35–37°C) and high relative humidity promote bacterial proliferation on fresh produce. In addition, the nutrient-rich tissues of cantaloupe and its reticulated texture provide a favorable microenvironment for *Salmonella* growth (24, 39).

A genomic expansion was observed between the two transconjugant *Salmonella* isolates following HGT on cantaloupe surfaces. In *S*. Typhimurium 14028 HGT, the acquisition of pSC27-derived sequences resulted in a 78.996 kb expansion compared to its wild-type strain (4,868,763 bp vs 4,789,767 bp, respectively). Sequence alignment confirmed the stable maintenance of the core backbone of the pSC27 plasmid in a reversed orientation. This large nucleotide increase suggests a complex cointegration event, where the mobilizable pSC27 backbone of the IncQ family triggered the cotransfer, transposition, or capture of larger mobile genetic elements, including fragments of the IncP RP4-8 helper plasmid, along with the native transposable machinery of the host (41–43).

In contrast, the BC-NL 5.2 HGT transconjugant exhibited a smaller genomic increase of 14.683 kb (expanding from 4,998,541 bp in the wild type to 5,013,224 bp in the transconjugant). Structural sequence analysis demonstrated that the core functional components of pSC27 (*bla*, *neo*, oriT) align with 100% identity within specific genomic coordinates, flanked by a ~6.1 kb accessory sequence block.

While both recipient strains successfully acquired and maintained the functional markers of antibiotic resistance (*bla*, *neo*) and the replication machinery (oriT) associated with the mobilizable pSC27 plasmid backbone (8,574 bp), the structural architecture and nucleotide scale of the acquired DNA differed significantly depending on the genomic background of the recipient.

We thoroughly evaluated the size discrepancy between the 8.574 kb pSC27 backbone and the total acquired region. This expansion of flanking regions during mobile element acquisition is a well-documented biological phenomenon rather than an assembly artifact. During the transposition of mobile elements or site-specific recombination events, the transfer machinery frequently triggers target site duplications (TSDs) and recruits flanking insertion sequences (IS elements) or transposases from the host or donor genome, expanding the nucleotide boundaries at the junction or insertion boundaries (43, 44). Furthermore, acquiring and stabilizing plasmid-derived resistance cassettes in enterobacterial genomes (including *Salmonella*) often involve local genomic rearrangements, target repeats, and the adjacent accumulation of mobile elements (44, 45). These results demonstrate that horizontal gene transfer on the surface of fresh produce is not uniform. Rather, it is determined by the intrinsic genomic plasticity of the recipient strain, the density of insertion sequences, and local recombination hotspots, resulting in varying degrees of accessory genome expansion in different *Salmonella* lineages.

In this study, the pSC27 plasmid was selected, as it has the RSF1010 region, which encodes relaxosomal components. Its transfer depends on the mating pair formation (Mpf) system of conjugative plasmids, facilitating the acquisition and/or introduction of its genome into host cells (46). As a member of the IncQ group, pSC27 is a significant vector for the dissemination of *bla* genes among various bacterial genera and species across diverse environments. These plasmids have been used as sources of replicons with a broad range of hosts in bacteria (41). They carry several ARGs that confer resistance to β-lactams, rendering them promiscuous and relevant in the spread of antimicrobial resistance genes. These genes are found in a wide range of hosts, where transfer, mobilization, and resistance genes are harbored (46, 47).

Although the pSC27 plasmid is not self-transferable, its mobilization by conjugative helper plasmids constitutes a significant mechanism for the dissemination of antimicrobial resistance genes. The transfer of pSC27 depends on the T4SS encoded by self-transferable plasmids such as RK2 and RP4 (41). For this reason, the RP4-8 plasmid was used as the helper plasmid for the transfer process. This plasmid has two transfer regions, Tra1 and Tra2, which encode the DNA transfer and mating pair formation functions required for conjugative transfer and enable the mobilization of pSC27 (46).

In this research, we demonstrated that all *Salmonella* isolates were capable of surviving and growing on the surface of melon at 35°C with 65–75% RH. Furthermore, all isolates were able to acquire the pSC27 plasmid from a donor strain of *E. coli* that possesses this conjugative plasmid, along with a helper plasmid. These studies were conducted both *in vitro* (using nutrient broth) and *in situ* (on the cantaloupe surface). *In vitro* was the most efficient method, as the bacteria were not subjected to stress and had the necessary environment for conjugation. However, on the surface of the cantaloupe, plasmid integration was possible, which is relevant and significant in their capacity for adaptation and evolution in different environments.

Mobilizable plasmids such as pSC27 are a growing concern due to their role in facilitating the horizontal transfer of antibiotic resistance genes between bacteria when co-resident with conjugative helper elements. Their efficient transfer to *Salmonella* on the surfaces of fresh produce underscores their potential relevance within the food chain. The presence of these mobilizable plasmids in food-related environments may contribute to the persistence, diversification, and dissemination of resistance genes among environmental and clinically relevant bacteria, emphasizing the need to monitor emerging mobilizable and conjugative plasmids.

The isolates studied are of utmost significance, as their phylogenetic analysis and percentage of similarity with the *Salmonella* enterica subspecies have confirmed their classification. Furthermore, upon placement on the surface of cantaloupe, numerous transformed cells were observed on the initial day of monitoring. This finding signifies a substantial risk for the rapid spread of antibiotic resistance and raises concerns regarding food safety. The present study highlights the remarkable adaptability, survival, and dissemination capabilities of *Salmonella* in environments such as cantaloupe, making a potential conduit for the transformation of multidrug-resistant bacteria.

The environmental conditions evaluated in this study likely favored HGT by conjugation on the cantaloupe surface. Conjugative plasmid transfer requires direct contact between donor and recipient cells; therefore, environmental conditions that promote bacterial survival, proliferation, and persistence increase the probability of successful transfer events. Consequently, the presence of these isolates in the environment and their potential for horizontal gene transfer across various ecological niches warrant meticulous attention.

### Conclusion

*Salmonella* can survive, proliferate, and acquire antibiotic resistance genes through HGT by incorporating them into its chromosome. This observation was made on cantaloupe surfaces in elevated ambient temperature (35°C) with RH 65–75% for 5 days. This observation underscores the potential risks associated with inadequate food storage temperatures, where the growth and transfer of genes between pathogens such as *Salmonella* and *E. coli* occur in foods. This impact extends beyond disease production and contributes to the rapid escalation of antimicrobial resistance and transmission of virulence genes, a significant threat to public health.

## Data Availability

This whole-genome shotgun project has been deposited in DDBJ/ENA/GenBank under BioProject accession number PRJNA1429812, with accession numbers JBYJEM010000000, JBYJEL010000000, JBYJEK010000000, and JBYJEJ010000000.

## SUPPLEMENTAL MATERIAL

Supplemental material aem.01076-26-s0001.docx Table S1, Fig. S1, and methodological details for phylogenetic reconstruction.

ASM does not own the copyrights to Supplemental Material that may be linked to, or accessed through, an article. The authors have granted ASM a non-exclusive, world-wide license to publish the Supplemental Material files. Please contact the corresponding author directly for reuse.
