# Supplementary material for: Survival, growth, and potential of horizontal gene transfer of antibiotic resistance genes of bovine Salmonella on the cantaloupe surface
Source: Appl Environ Microbiol. 2026 Oct 1;92(10):e01076-26. doi: 10.1128/aem.01076-26 (PMC13689264; doi:10.1128/aem.01076-26)
Supplement: Supplementary file 1 [file aem.01076-26-s0001.docx]

Table S1. Comprehensive genomic validation and sequencing statistics for *Salmonella* isolates based on the 3C criteria (Molina-Mora et al., 2020).

| Quality Category / Parameter | S. Typhimurium 14028 | S. Typhimurium 14028 HGT | BC-NL 5.2 | BC-NL 5.2 HGT | Software / Pipeline Tool | Verification & Biological Significance |
| --- | --- | --- | --- | --- | --- | --- |
| Basecaller / Algorithm | Dorado (v1.1.0) | Dorado (v1.1.0) | Dorado (v1.1.0) | Dorado (v1.1.0) | ONT Dorado | Real-time high-accuracy basecalling workflow. |
| Workflow Suite | wf-bacterial-genomes (v1.4.2) | wf-bacterial-genomes (v1.4.2) | wf-bacterial-genomes (v1.4.2) | wf-bacterial-genomes (v1.4.2) | EPI2ME labs | Standardized Nextflow pipeline for ONT bacterial genomes. |
| Min. Read Length Cutoff | 1,000 bp | 1,000 bp | 1,000 bp | 1,000 bp | wf-bacterial-genomes | Filters out short DNA fragments to preserve long-read assembly integrity. |
| Genome Read Depth / Coverage (GenBank Declared) | 55X | 60X | 75X | 40X | GenBank / NCBI Submission | High-depth uniform sequencing read depth across the entire genome. |
| Mean Read Quality (Q-score) | 10.2 | 10.5 | ~11.5 | 10.2 | NanoPlot (v1.46.1) / fastcat (v0.22.0) | Demonstrates high-accuracy raw base calls. |
| Median Read Quality (Q-score) | 10.3 | 10.7 | ~11.7 | 10.5 | NanoPlot (v1.46.1) / fastcat (v0.22.0) | Robust statistical distribution of read quality. |
| Mean Read Length | 3,284.1 bp | 3,028.0 bp | ~5,250.3 bp | 3,222.7 bp | NanoPlot (v1.46.1) / fastcat (v0.22.0) | High average read length to span repeat regions. |
| Median Read Length | 1,793.0 bp | 1,529.0 bp | ~3,571.0 bp | 1,737.0 bp | NanoPlot (v1.46.1) / fastcat (v0.22.0) | Consistent long-read length across the sequencing run. |
| Max Read Length | 57,213 bp | 79,155 bp | N/A | 57,406 bp | NanoPlot (v1.46.1) / fastcat (v0.22.0) | Maximum single continuous read length captured. |
| Consensus Polishing | Medaka workflow | Medaka workflow | Medaka workflow | Medaka workflow | Medaka (v2.0.0) / pomoxis (v0.3.16) | Neural-network consensus correction to resolve indels. |
| De Novo Assembler | Flye (v2.9.5-b1801) | Flye (v2.9.5-b1801) | Flye (v2.9.5-b1801) | Flye (v2.9.5-b1801) | Flye assembler | Algorithmic assembly optimized for long, uncorrected reads. |
| Total Assembly Size | 4,815,512 bp | 4,863,530 bp | 4,993,337 bp | 5,008,612 bp | Flye (v2.9.5-b1801) | Sum of all resolved physical contigs. |
| N50 Metric | 4,717,888 bp | 4,716,876 bp | 4,895,560 bp | 4,900,199 bp | Flye (v2.9.5-b1801) | Represents the high-contiguity chromosomal backbone. |
| %GC Content | 52% | 52% | 52% | 52% |  |  |
| Number of Contigs | 2 | 4 | 3 | 3 | Flye (v2.9.5-b1801) | Complete structural resolution of the genome. |
| Contig Circularization | 100% (2/2 Circular) | 100% (4/4 Circular) | 100% (3/3 Circular) | 100% (3/3 Circular) | Flye (v2.9.5-b1801) | Terminal repeats resolved; all molecules are circular. |
| Chromosomal Contig Size | 4,717,888 bp (contig_2) | 4,716,876 bp (contig_1) | 4,895,560 bp ( contig_1) | 4,900,199 bp (contig_1) | Flye (v2.9.5-b1801) | Chromosome size. Circular: YES (Y), Repeat: NO (N), Multiplicity: 1. |
| Plasmid 1 Contig Size | 97,624 bp (contig_1) | 82,050 bp (contig_4) | 91,389 bp (contig_1) | 91,348 bp (contig_3) | Flye (v2.9.5-b1801) | Large plasmid size. Circular: Y, Repeat: N, Multiplicity: 1 (2 for Strain 4). |
| Plasmid 2 Contig Size | N/A (Absent) | 67,552 bp (contig_2) | 6,388 bp (contig_3) | 17,065 bp (contig_2) | Flye (v2.9.5-b1801) | Mid/small plasmid size. Circular: Y, Repeat: Y, Multiplicity: 1 (2 for Strain 4). |
| Plasmid 3 Contig Size | N/A (Absent) | 17,052 bp (contig_3) | N/A (Absent) | N/A (Absent) | Flye (v2.9.5-b1801) | Small plasmid size. Circular: Y, Repeat: Y, Multiplicity: 4. |
| Total CDC/ Protein-cogind Genes | 4,845 genes | 4,978 genes | 5,065 genes | 5,810 genes | Prokka (v1.15.6) | Fully covers the expected biological and metabolic gene space. |
| MLST Typing Tool | mlst (v2.23.0) | mlst (v2.23.0) | mlst (v2.23.0) | mlst (v2.23.0) | mlst / pysam (v0.22.0) | Standard sequence typing confirming *Salmonella* lineage. |
| ResFinder Thresholds | Identity: 0.8 / Cov: 0.6 | Identity: 0.8 / Cov: 0.6 | Identity: 0.8 / Cov: 0.6 | Identity: 0.8 / Cov: 0.6 | ResFinder / wf-bacterial-genomes | Hardcoded parameters to confirm resistance marker completeness. |

Figure S1 Phylogenetic tree depicting the evolutionary relationships among *Salmonella* species based on *16S* *rRNA* and *RecA* genes. The tree illustrates the genetic relationships among different genomes and their positions within the evolutionary lineage. Bootstrap values derived from 1,000 replicates, spanning from 45 (lowest) to 100 (highest) provide statistical validation for the inferred relationships.

Supplementary text: Methodological details for phylogenetic reconstruction.

To determine the genetic positioning and taxonomic placement of the *Salmonella* isolates and transconjugants recovered from cantaloupe, a phylogenetic analysis was conducted using concatenated *16sRNA* and *recA* gene sequences. These conserved genetic markers were selected because they provide complementary phylogenetic resolution and are widely used for bacterial taxonomic. Sequence alignments and phylogenetic reconstruction were performed in MEGA12 (1). Phylogenetic reconstruction was inferred using the maximum verisimilitude method based on the Tamura-Nei substitution model with a gamma distribution and invariant sites (TN93+G+I), employing the Nearest-Neighbor-Interchange (NNI) heuristic search algorithm. Branch support and tree reliability were evaluated using bootstrap analysis with 1,000 replicates. Reference sequences of *Salmonella enterica* were retrieved from GenBank for comparison.

References:

1. Kumar S, Stecher G, Suleski M, Sanderford M, Sharma S, Tamura K. 2024. MEGA12: Molecular Evolutionary Genetic Analysis version 12 for adaptive and green computing. Mol Biol Evol 41:msae263.0
